# Effect modification by geographic area on the association between health literacy and self-rated health: a nationwide cross-sectional study in Japan

**DOI:** 10.1186/s12889-023-15897-0

**Published:** 2023-05-25

**Authors:** Hirono Ishikawa, Runa Ogawa, Aki Otsuki, Junko Saito, Akiko Yaguchi-Saito, Aya Kuchiba, Maiko Fujimori, Yoshiharu Fukuda, Taichi Shimazu, INFORM Study Group, Masayo Hayakawa, Satoyo Hosono, Manami Inoue, Yuki Kaji, Kota Katanoda, Tomohiro Matsuda, Yutaka J. Matsuoka, Miyuki Odawara, Hirokazu Takahashi, Miyako Takahashi, Yosuke Uchitomi, Jun Umezawa, Otome Watanabe, Itsuro Yoshimi, Gary L. Kreps, Naomi Sakurai, Takumi Momosaka, Miki Akiyama, Rina Miyawaki, Ryo Okubo, Rie Akamatsu, Yui Kawasaki, Kahori Fujisaki, Luna Ogawa, Haruhiko Imamura, Kumi Nakaya, Naoki Nakaya, Seigo Mitsutake, Hiroyuki Sasai

**Affiliations:** 1 Teikyo University Graduate School of Public Health, 2-11-1 Kaga, Itabashi-Ku, Tokyo, 173-8605 Japan; 2 Division of Behavioral Sciences, National Cancer Center Institute for Cancer Control, National Cancer Center, 5‐1‐1 Tsukiji, Chuo‐ku, Tokyo, 104‐0045 Japan; 3 Faculty of Human Sciences, Tokiwa University, 1-430-1 Miwa, Mito-Shi, Ibaraki, 310-8585 Japan; 4 Graduate School of Health Innovation, Kanagawa University of Human Services, 3-25-10 Tonomachi, Kawasaki-Ku, Kawasaki-Shi, Kanagawa, 210-0821 Japan; 5 Division of Biostatistical Research, Institute for Cancer Control/Biostatistics Division, Center for Research Administration and Support, National Cancer Center, 5‐1‐1 Tsukiji, Chuo‐ku, Tokyo, 104‐0045 Japan; 6 Division of Survivorship Research, National Cancer Center Institute for Cancer Control, National Cancer Center, 5‐1‐1 Tsukiji, Chuo‐ku, Tokyo, 104‐0045 Japan

**Keywords:** Health literacy, Geographic differences, INFORM Study, Japan, Self-rated health

## Abstract

**Background:**

Health literacy (HL) has gained increasing attention as a factor related to health behaviors and outcomes. This study aimed to investigate geographic differences in HL levels and effect modification by geographic area on their relationship with self-rated health in the Japanese population using a nationwide sample.

**Methods:**

Data for this study were derived from a nationally representative cross-sectional survey on health information access for consumers in Japan using a mailed self-administered questionnaire in 2020 (INFORM Study 2020). Valid responses from 3,511 survey participants, selected using two-stage stratified random sampling, were analyzed in this study. HL was measured using the Communicative and Critical Health Literacy Scale (CCHL). Multiple regression and logistic regression analyses were conducted to examine the associations between geographic characteristics and HL and effect modification on the association between HL and self-rated health by geographic area, controlling for sociodemographic characteristics.

**Results:**

The mean HL score was 3.45 (SD = 0.78), somewhat lower compared with previous studies on the Japanese general population. HL was higher in Kanto area than in Chubu area, after controlling for sociodemographic factors and municipality size. Furthermore, HL was positively associated with self-rated health after controlling for sociodemographic and geographic factors; however, this association was more evident in eastern areas than in western areas.

**Conclusion:**

The findings indicate geographic differences in HL levels and effect modification by geographic area on the relationship between HL and self-rated health in the general Japanese population. HL was more strongly associated with self-rated health in eastern areas than in western areas. Further investigation is needed to explore the moderating effects of areal features, including the distribution of primary care physicians and social capital, when formulating strategies to improve HL in different contexts.

**Supplementary Information:**

The online version contains supplementary material available at 10.1186/s12889-023-15897-0.

## Background

Over the past few decades, health literacy (HL) has gained increasing attention as a factor related to health behaviors and outcomes. Although its definition and operationalization are still evolving [1–3], existing definitions of HL have similar core elements in that they describe the personal skills that enable individuals to obtain, understand, and use information to make decisions and take actions that will impact their health [2]. Inadequate or limited HL is related to lower self-rated health, negative health outcomes, higher healthcare costs, and lower quality of care [4, 5].

Research on HL has gradually moved beyond a focus on the individual and toward the interaction between the demands of health systems and the skills of individuals [6, 7]. HL is mediated by organizational structures and the availability of resources that enable people to access, understand, appraise, and use information and services in ways that promote and maintain good health, not only for themselves but also for those around them [8]. Furthermore, resources and support provided through social networks have been found to buffer and alleviate the adverse consequences of inadequate individual HL [9, 10]. Thus, HL is not solely an individual skill but a distributed resource available within an individual’s social environment. HL-related problems in society can be resolved by developing the skills of individuals and by lowering the barriers created by health service personnel and systems [11]. To this end, it is necessary to examine factors related to HL at the areal level.

A previous study in Hungary reported no significant difference in HL levels by geographical residence; however, the magnitude of the effect of social support on HL was stronger in rural areas [12]. A study in China found heterogeneity in HL among different areas, between urban and rural areas, and among different social groups [13]. A systematic review encompassing several countries reported that HL differences exist between rural and urban populations, although living in a rural area is not the only reason for HL disparities [14]. Social and cultural factors could be different among areas even within the same nation, which may be related to utilization of health information and services. Given such geographic differences, tailored health communication strategies, such as selecting appropriate media and collaborating with available healthcare resources, might be needed to improve HL. Further, exploring areal differences in the relationship between HL and health outcomes may reveal key social environmental characteristics that interact with individual HL.

There is a growing research interest in HL in the Asian context, and nationwide surveys have been conducted in countries such as South Korea and Taiwan, among others [15–17]. In Japan, several studies have used nationwide community-based surveys to examine the relationship between municipality size, sociodemographic factors, and HL. Studies using a research company’s database (Internet survey or mail survey) reported that female gender, older age, and higher income were associated with higher HL [18, 19]. Whereas, nationally representative surveys using stratified random sampling reported that higher HL was associated with younger age [20, 21], higher educational attainment, higher income, and managerial occupations [20]. In addition, these studies suggested that a greater municipality size was associated with higher HL, and higher HL was associated with better self-rated health [20, 21]. Self-rated health is a reliable predictor of objective health outcomes, such as mortality [22, 23], and has been widely used in public health studies. Moreover, previous studies in Japan have suggested geographical disparities in healthcare resources and their association with health outcomes [24, 25]. However, few studies have examined geographic variation in HL using validated measures and controlling for sociodemographic variables or the moderating effect of geographic areas on the relationship between HL and health outcomes.

Thus, this study aimed to investigate geographic differences in HL levels and effect modification by geographic area on their relationship with self-rated health in the Japanese population using a nationwide sample. Specifically, we aimed to:

1. Identify HL levels, measured using the Communicative and Critical Health Literacy Scale (CCHL), in the general Japanese population and compare these with previous studies in Japan that used the same scale.
2. Explore geographic differences in HL controlling for sociodemographic factors.
3. Examine whether HL is associated with self-rated health and whether the association is moderated by geographical area.

## Methods

### Study design and participants

**Fig. 1:**
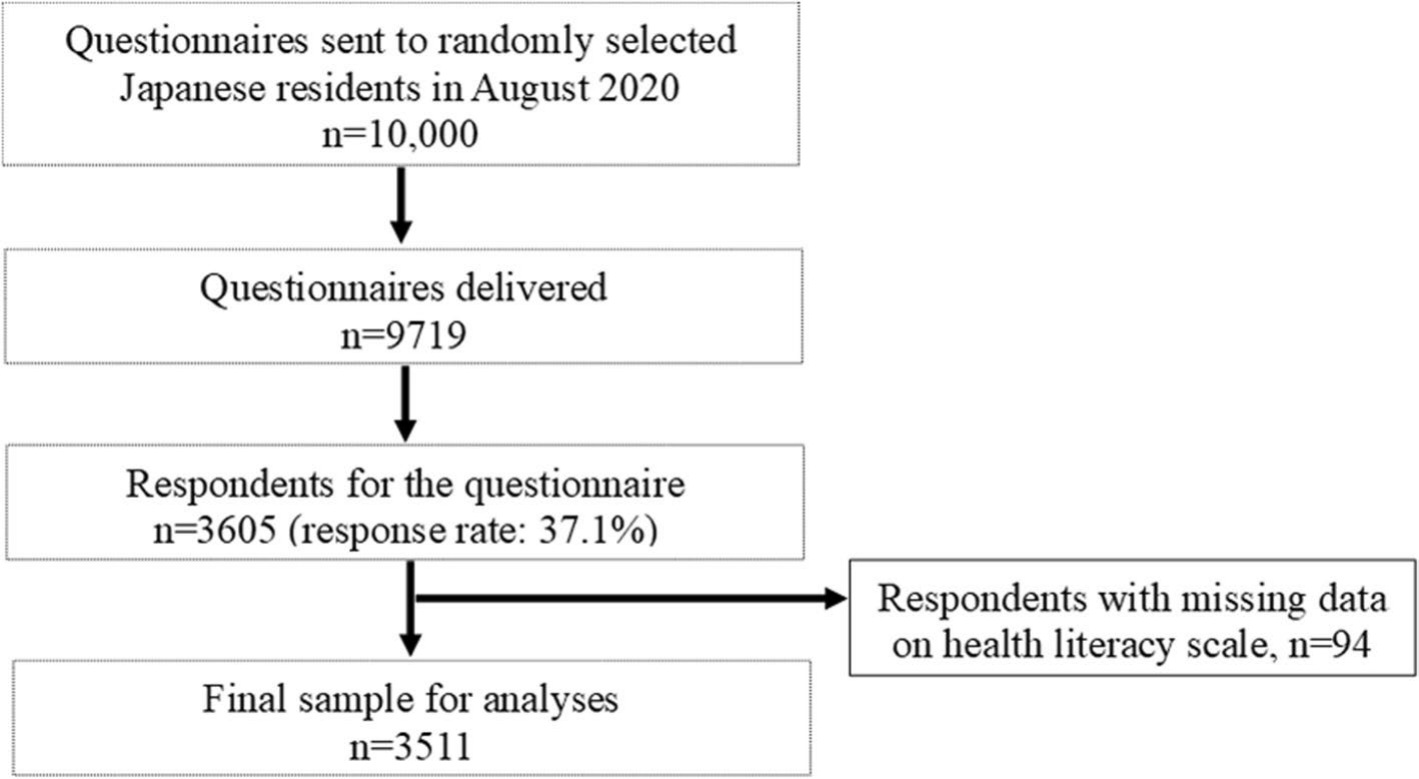
Flow chart of participant selection in the INFORM Study 2020

Data for this study were derived from a nationally representative cross-sectional survey on health information access for consumers in Japan (INFORM Study) [26] using a mailed self-administered questionnaire in 2020 (INFORM Study 2020). While the INFORM Study 2020 was designed to investigate consumer behaviors related to cancer prevention and screening, the survey also collected variables on general health information access and utilization, such as HL, communication with healthcare professionals, and internet use. The details of the sampling strategy used for this survey have been described in the protocol paper [26]. Briefly, 10,000 Japanese individuals were sampled using two-stage stratified random sampling, with the census area as the primary sampling unit and individuals aged 20 years or older as the secondary sampling unit. From 35 strata, by crossing nine areas and four municipality groups by population size, we randomly selected 500 census areas with probability proportional to the size of the stratum. The self-administered questionnaire consisted of core items from the Health Information National Trends Survey (HINTS) in the United States [27], with some additional items not covered in the HINTS but important in Japan. As shown in Fig. 1, Data collection for the INFORM Study 2020 began on August 1, 2020, and concluded on September 30, 2020, with a total of 3,605 participants completing the survey (Fig. 1). After excluding those with missing data on the HL scale items, 3,511 participants were included in the present analysis.

### Measurements

#### Health literacy

HL was measured using the Communicative and Critical Health Literacy Scale (CCHL) [28], developed in Japan based on an established model of HL [29], and used in many studies to assess respondents’ perceived ability to find and use health and medical information as required. It consists of five items assessing whether the respondent is able to 1) collect information from various sources, 2) extract the information needed, 3) understand and communicate the obtained information, 4) judge the credibility of the information, and 5) make decisions about actions and plans for health improvement based on the information. Each item is rated on a five-point scale ranging from strongly disagree (1) to strongly agree (5). The scores for all items were summed and divided by the number of items in the scale to yield a scaled score (theoretical range: 1 − 5) [28]. The Cronbach’s alpha for the scale was 0.87.

#### Self-rated health

Self-rated health was measured by the following question: “In general, would you say your health is 1) excellent, 2) very good, 3) good, 4) fair, or 5) poor?” In the analyses, responses 1), 2), and 3) were coded as 1, indicating good health.

#### Sociodemographic variables

Data for the following demographic and socioeconomic variables were also obtained as part of the survey: age (in years), gender (male or female), educational attainment (junior high school, high school, vocational school or 2-year college, or university and above; used as order variable), household income (less than 2, 2 − 4, 4 − 6, 6 − 8, 8 − 10, or more than 10 million yen; used as order variable), marital status (married or not currently married [never married, divorced, or widowed]), and employment status (employed in a managerial or professional position, employed as other, or not employed [homemaker, student, unemployed, or retired]).

#### Geographic variables

The area of residence was coded as Hokkaido/Tohoku (northern part), Kanto (the eastern part where Tokyo, the capital of Japan, is located), Chubu (central part), Kinki (the south-central part where Osaka, the largest city in the western area, is located), Chugoku/Shikoku (western part), and Kyusyu (southwestern part). To examine the effect modification, the areas were divided into eastern (the former three) and western (the latter three) areas. The division of the East and West is routinely used in Japanese society, and many differences between the areas are acknowledged, including culture and customs.

Municipality size was categorized into the following: 21 major cities with a population of 500,000 or more, large cities with a population of 200,000 or more, smaller cities with a population of less than 200,000, and towns and villages.

### Statistical analyses

We conducted a weighted analysis to account for the complex sampling design and nonresponses in order to calculate accurate population parameter estimates for the Japanese general population. The weight for each participant was calculated by multiplying the sampling weight and the nonresponse weight. Based on the sampling strategy of the survey, the sampling weight for each participant was calculated as the reciprocal of the probability of selecting the participant for the survey in the stratum. The nonresponse weight was estimated as the reciprocal of the proportion of respondents in “nonresponse adjustment cells”, assuming that the respondents in each nonresponse adjustment cell are a random sample of the samples in that cell [30]. We used a Search algorithm [31] to create the nonresponse adjustment cells based on the variables of the sampling strata, gender, and age group (20–29, 30–39, 40–49, 50–59, 60–69, 70–79, 80 or more), resulting in a total of 26 nonresponse adjustment cells. The confidence intervals (CI) were estimated using the Taylor series linearization method [32]. All analyses were performed using STATA (version 17.0) svyset command with 500 census areas as the variable of the primary sampling units and the 35 sampling strata as the variable of strata.

Multiple regression analysis was conducted to examine the association between geographic characteristics and HL, controlling for sociodemographic factors. Logistic regression analysis was used to examine the association between HL and self-rated health, controlling for sociodemographic and geographic characteristics. Stratified analyses by area were also conducted to explore effect modification by geographic area on this association. Results with a p-value < 0.05 were deemed statistically significant.

### Ethical approval

The INFORM Study 2020 protocol was approved by the Research Ethics Committee of the National Cancer Center (research project number: 2019–290) and Teikyo University (research project number: 20–211).

## Results

### Participants’ characteristics and descriptive statistics

Table 1 summarizes the participants’ sociodemographic and geographic characteristics. The mean age was 54.3 years (95% CI: 53.6 − 55.1), and 49.3% of the participants were male. Based on the weighted analyses, 30.4% of the participants had graduated from university and above, and 66.9% were currently married. The annual household income was 2 − 4 million yen for 26.4% of the respondents and 4 − 6 million yen for 22.1%. The proportion of those employed in managerial and professional positions was 23.2%, while 36.6% were currently not employed, including homemakers, students, and unemployed and retired persons. Overall, 2,681 (76.1%) participants had good self-rated health.

**Table 1:** Respondent characteristics and health literacy for INFORM Study 2020

		Unweighted		Weighted		Health literacy	
		N	%	%		Mean	95%CI
**Sociodemographic characteristics**							
**Age**	20–39	692	19.7	23.9		3.52	[3.46–3.57]
	40–54	969	27.6	26.8		3.61	[3.57–3.65]
	55–69	996	28.4	23.9		3.51	[3.47–3.56]
	70-	854	24.3	25.4		3.17	[3.10–3.24]
	Mean age [95%CI]			54.3 [53.6–55.1]			
**Gender**	Male	1599	45.5	49.3		3.46	[3.42–3.50]
	Female	1912	54.5	50.7		3.45	[3.40–3.49]
**Educational attainment**	Junior high school	282	8.0	8.8		2.86	[2.73–2.99]
	High school	1368	39.0	38.6		3.39	[3.34–3.43]
	Vocational school/2-year college	819	23.3	21.9		3.54	[3.49–3.58]
	University and above	1031	29.4	30.4		3.64	[3.60–3.68]
	missing	11	0.3	0.3			
**Household income** (yen)	Less than 2 million	326	9.3	9.7		3.11	[2.98–3.23]
	2–4 million	948	27.0	26.4		3.38	[3.33–3.44]
	4–6 million	777	22.1	21.8		3.48	[3.42–3.53]
	6–8 million	571	16.3	16.3		3.54	[3.49–3.60]
	8–10 million	366	10.4	10.7		3.58	[3.50–3.66]
	More than 10 million	423	12.0	12.1		3.67	[3.60–3.74]
	missing	100	2.8	3.0			
**Marital status**	Married	2456	70.0	66.9		3.49	[3.46–3.52]
	Never married	597	17.0	19.4		3.50	[3.44–3.56]
	Divorced	242	6.9	8.1		3.09	[2.94–3.24]
	Widowed	205	5.8	5.4		3.37	[3.26–3.47]
	missing	11	0.3	0.3			
**Employment status**	Employed (Managerial and professional)	800	22.8	23.2		3.67	[3.63–3.72]
	Employed (other)	1424	40.6	40.2		3.48	[3.44–3.52]
	Not employed (homemaker/ student/ unemployed/ retired)	1281	36.5	36.6		3.28	[3.23–3.34]
	missing	6	0.2				
**Geographic characteristics**							
**Area**	Hokkaido/Tohoku area	394	11.2	11.6		3.41	[3.29–3.54]
	Kanto area	1,187	33.8	33.3		3.52	[3.47–3.56]
	Chubu area	686	19.5	18.4		3.37	[3.31–3.42]
	Kinki area	552	15.7	16.2		3.47	[3.41–3.54]
	Chugoku/Shikoku area	318	9.1	9.2		3.40	[3.33–3.48]
	Kyusyu area	374	10.7	11.3		3.45	[3.35–3.56]
**Municipality size**	21 major cities	1020	29.1	28.8		3.54	[3.48–3.59]
	Other large cities	819	23.3	23.0		3.45	[3.38–3.51]
	Smaller cities	1375	39.2	40.0		3.42	[3.37–3.46]
	Towns and villages	297	8.5	8.3		3.34	[3.24–3.44]
**Self-rated health**	Excellent	114	3.2	3.7		3.78	[3.63–3.93]
	Very good	577	16.4	16.8		3.71	[3.65–3.77]
	Good	1990	56.7	55.6		3.47	[3.44–3.51]
	Fair	745	21.2	21.2		3.21	[3.14–3.28]
	Poor	82	2.3	2.5		2.92	[2.64–3.19]
	missing	3	0.1	0.1			

### HL by sociodemographic and geographic factors

The mean score of HL was estimated at 3.45 (SD = 0.78, 95% CI: 3.42 − 3.48; Table 2). The majority of the participants reported that they had the ability to collect information from various sources (80.3%), whereas less than half agreed that they had the ability to judge the credibility of the information (44.7%).

**Table 2:** Distribution of health literacy scale items (weighted)

			*N* = 3511		
	N^a^	%^a^	Mean	SD	95%CI
To collect information from various sources	2850	80.3	3.85	0.94	[3.82–3.89]
To extract the information needed	2243	63.1	3.50	0.98	[3.46–3.53]
To understand and communicate the obtained information	1844	51.8	3.32	0.96	[3.29–3.36]
To judge the credibility of the information	1575	44.7	3.23	0.92	[3.20–3.26]
To make decisions based on the information	1928	54.3	3.36	0.96	[3.32–3.40]
Total scale			3.45	0.78	[3.42–3.48]

^a^The number and percentage of the participants who agreed/strongly agreed that they had the ability

Differences in the participants’ HL status according to sociodemographic and geographic factors are shown in Table 1. The relationships between geographic factors and HL were examined using multiple regression analysis (Table 3). Higher HL was associated with being under 70 years of age, female gender, higher educational attainment, higher household income, being married, and being employed in managerial and professional positions. Controlling for these sociodemographic factors, living in Chubu area was associated with lower HL, compared with Kanto.

**Table 3:** Relationships of geographic and sociodemographic factors with health literacy

	Regression coefficient	s.e	p-value
**Age** (ref: 70-)			
20–39	**0.173**	**0.051**	**0.001**
40–54	**0.225**	**0.047**	** < 0.001**
55–69	**0.198**	**0.045**	**< 0.001**
**Gender** (ref: male)	**0.064**	**0.027**	**0.017**
**Educational attainment**	**0.124**	**0.016**	**< 0.001**
**Household income**	**0.032**	**0.011**	**0.003**
**Marital status** (ref: not currently married)	**0.067**	**0.034**	**0.047**
**Employment status** (ref: Not employed)			
Employed (Managerial and professional)	**0.117**	**0.039**	**0.003**
Employed (other)	0.032	0.035	0.373
**Municipality size** (ref: Smaller cities)			
21 major cities	0.028	0.034	0.414
Other large cities	-0.020	0.039	0.609
Towns and villages	-0.063	0.054	0.247
**Area** (ref: Kanto)			
Hokkaido/Tohoku	-0.022	0.060	0.710
Chubu	**-0.110**	**0.039**	**0.005**
Kinki	-0.003	0.039	0.945
Chugoku/Shikoku	-0.051	0.044	0.241
Kyusyu	-0.005	0.055	0.925
Constant	2.650	0.089	< 0.001
R-squared	0.091		

Multivariable linear regression analysis was used. Significant differences are printed in bold (*p* < 0.05)

### Effect modification by geographic area on the relationship

The relationship between HL and self-rated health was examined using logistic regression analysis, controlling for sociodemographic and geographic factors. As shown in Table 4, higher HL was significantly associated with better self-rated health. The stratified analyses by area suggested that this association was more evident in eastern areas (Hokkaido/Tohoku, Kanto, and Chubu), but not significant in western areas (Kinki, Chugoku/Shikoku, and Kyusyu). Based on this, the significance of the effect modification was examined by dividing the areas to eastern and western areas as shown in Table 5. The interaction between HL and these areas (eastern areas versus western areas) was statistically significant.

**Table 4:** Adjusted odds ratio of health literacy with self-rated health by geographic area

	N	OR	95%CI
**Total sample**^a^	3394	**1.531**	**[1.358–1.727]**
**By area**^b^			
Hokkaido/Tohoku	376	**1.748**	**[1.208–2.530]**
Kanto	1152	**1.630**	**[1.300–2.043]**
Chubu	657	**1.731**	**[1.351–2.217]**
Kinki	539	1.216	[0.898–1.647]
Chugoku/Shikoku	307	1.366	[0.916–2.038]
Kyusyu	363	1.489	[1.000–2.216]

Significant differences are printed in bold (*p* < 0.05)

^a^OR is from logistic regression analysis adjusted for age, gender, education, household income, marital status, employment status, municipality size, and area

^b^OR is from logistic regression analysis adjusted for age, gender, education, household income, marital status, employment status, and municipality size

**Table 5:** Effect modification by geographic area on the association between health literacy and self-rated health

	OR^1^	95% CI
**Municipality size** (ref: Smaller cities)		
21 major cities	0.847	[0.684–1.049]
Other large cities	0.905	[0.734–1.116]
Towns and villages	0.937	[0.656–1.339]
**Western areas** (ref: Eastern areas)	2.096	[0.909–4.834]
**Health literacy**	**1.685**	**[1.450–1.958]**
**Areas×Health literacy**	**0.776**	**[0.608–0.990]**

Significant differences are printed in bold (*p* < 0.05)

^1^Multiple logistic regression analysis controlling for age, gender, educational attainment, household income, marital status, and employment status

## Discussion

This study examined geographic differences in HL levels in the general Japanese population using a validated HL measure and a nationwide sample and investigated effect modification by geographic area on their relationship with self-rated health. We conducted a weighted analysis to account for the complex sampling design and missing responses. The mean HL score, measured using the CCHL scale, was 3.45, somewhat lower compared with previous studies on the Japanese general population (Supplementary Table 1); for example, a mean score of 3.61 (SD = 0.75) was reported by a nationwide survey using the mail-placement method with random sampling (*N* = 2037) [21], a score of 3.61 (SD = 0.64) was reported by another study using mail survey with the database of a survey research company (*N* = 1002) [33], a score of 3.59 (SD = 0.62) was reported by a study using an Internet-based survey and the database of a survey research company (*N* = 713) [34], and a score of 3.63 (SD = 0.64) was reported by another study in the Tokyo metropolitan area (*N* = 3663) [35]. The lower score obtained in our study may be partly because it included participants aged over 80 years, not included in the previous studies. Indeed, the mean HL score was estimated at 3.51 after excluding participants aged 80 years and above, although it was still relatively low.

Another possible reason for the low score is the influence of the COVID-19 pandemic, as the survey was conducted in 2020. A previous longitudinal study reported a significant decline in HL during the first year of the pandemic [36]. Several problems related to health communication emerged during the pandemic. In particular, false or contradictory information spread rapidly via social media and other Internet outlets, and the “infodemic” (the global epidemic of misinformation) posed a serious problem for public health [37]. Indeed, another part of our survey (INFORM Study 2020) showed that 58.5% of the participants sought information about COVID-19 every day during the month this survey was conducted, and 83.1% were concerned about COVID-19. A lower HL score may reflect difficulties in obtaining and understanding adequate information in the context of the COVID-19 pandemic. As reported in a previous study [28], the majority of the participants stated that they had the ability to collect information from various sources, whereas, the proportion of those who stated that they had the ability to judge the credibility of the information was particularly low in this study. While access to information may have become easier with the widespread use of the Internet and smartphones, judging the quality of the information to make decisions may still pose difficulties. Hence, educational programs on the proper use of online information may be required to improve HL. Further research is needed in the future to confirm HL levels in Japan.

### Geographic differences in HL

The associations between sociodemographic factors and HL were generally consistent with previous nationwide studies in Japan [18–21]. Higher HL was associated with being under 70 years of age, female gender, higher educational attainment, higher household income, being married, and being employed in managerial and professional positions. Notably, the association between age and HL status may not be linear. In this study, those aged 70 years and older had significantly lower HL than the other age groups, while the gradient in HL among those aged 20 − 69 years was unclear. This may partly explain the inconsistent findings regarding the relationship between age and HL among previous studies [18–21].

Although HL tended to be higher in larger cities, as also reported by bivariate analyses in previous studies [19–21], a significant difference by municipality size was not found after controlling for these sociodemographic factors. This was mainly because there were a greater proportion of younger people and people with higher educational attainment in the larger cities. By contrast, HL scores were lower in Chubu area compared with Kanto, after controlling for these sociodemographic factors and municipality size. Kanto is the most urbanized area in the eastern areas of Japan, where Tokyo, the capital of Japan, is located. Many higher education institutions and major companies are located here, and Internet usage rates tend to be high (Ministry of Internal Affairs and Communications, Communications Usage Trend Survey, 2020). Another part of INFORM Study 2020 showed that the proportion of those who accessed the Internet or World Wide Web was higher in the Kanto (82%) than in other areas (Hokkaido/Tohoku: 70.6%; Chubu: 77.4%; Kinki: 79.9%; Chugoku/Shikoku: 77.2%; Kyusyu: 77.3%; data not shown). It is plausible that Kanto area provides better Internet infrastructure, more extensive health information services, and more educational opportunities to improve HL. Even those who do not directly use these services may benefit from having more individuals with higher HL in the community, who could help them access and utilize health information and services. By contrast, the reason for low HL in Chubu area is unclear. This area has a high concentration of manufacturing industries, and the main city in this area, Nagoya, is known for its somewhat closed and conservative culture, despite being the fourth largest city in Japan [38]. These cultural characteristics of the area may be associated with less active information-seeking and decision-making, and thus, lower HL. Interestingly, however, several prefectures in Chubu area are known for higher life expectancy. Despite the lower HL, self-rated health was not lower in Chubu than it was in Kanto. Further research is needed to investigate geographic differences in HL and related factors.

### Effect modification by geographic area on the relationship

Consistent with previous studies, HL was associated with better self-rated health after controlling for sociodemographic and geographic factors. However, the association between HL and self-rated health differed by geographic area. HL was more strongly associated with self-rated health in eastern areas (Hokkaido/Tohoku, Kanto, and Chubu) than in western areas (Kinki, Chugoku/Shikoku, and Kyusyu).

One possible explanation for this finding may be the distribution of primary care physicians. Reports have shown that the number of medical institutions with primary care functions per 100,000 persons is generally higher in the West than in the East, in Japan [39, 40]. An important feature of the Japanese healthcare system is “free access.” Patients are free to choose any healthcare facility regardless of the severity of their disease and their insurance status [24, 41]. Ironically, a previous study showed that a greater proportion of Japanese residents found it difficult to know where to obtain professional help when they were ill compared with European residents [19]. Lower HL is associated with poor patient access to and coordination of care [42]. Unrestricted access to any doctor without a usual source of care can make it difficult to find appropriate healthcare services, especially for patients with low HL. Primary care is regarded as a setting that can potentially mitigate HL inequalities among patients [43]. Easier accessibility to primary care may help moderate the relationship between low HL and low health status.

Similarly, it has been reported that social capital tends to be higher in prefectures in the western area of Japan [44]. Social capital is the resources available to individuals and groups through their membership in social networks [45]. Previous studies have suggested that valuable resources and support from one’s social networks buffer and alleviate the adverse consequences of low HL [46, 47]. HL is mediated by organizational structures and the availability of resources that enable people to access, understand, appraise, and use information and services in ways that promote and maintain good health for themselves and those around them [8]. Although we could not directly examine this hypothesis in this study, greater social capital may be a potential factor that moderates the relationship between HL and health status in the western area of Japan compared with the eastern area. Further investigation is needed to explore the moderating effects of areal features, including access to primary care and social capital, on the relationship between HL and health outcomes. The results can help formulate strategies to improve HL in different contexts.

### Limitations and agenda for future research

This study has several limitations. First, the original survey had a response rate of 37%. Although the rate was comparable to that of the HINTS survey in the United States and was adjusted for non-response bias by weighting, the generalizability of the current findings might be limited. Second, the survey was conducted during the first year of the COVID-19 pandemic, which profoundly impacted people’s health and daily lives. Thus, our results may be specific to the present study. However, the associations between HL and sociodemographic factors were generally similar to those reported in previous studies. Third, the self-administration of questionnaires requires at least a basic literacy level, which may have biased the findings to some degree. Fourth, HL status and health status were measured using self-report items. The responses represented the participants’ perceptions and may differ from their objective ability and health. Although many self-reported measures of HL, including the CCHL, have been widely used and validated in previous studies, further research using objective measures is required to confirm the relationships found in this study.

## Conclusions

This study examined geographic differences in HL levels and effect modification by geographic area on the relationship between HL and self-rated health in the general Japanese population using a nationwide sample. After controlling for sociodemographic factors and municipality size, HL was higher in Kanto area than in Chubu area. Furthermore, HL was associated with better self-rated health after controlling for sociodemographic and geographic factors; however, this association was more evident in eastern areas (Hokkaido/Tohoku, Kanto, and Chubu) than in western areas (Kinki, Chugoku/Shikoku, and Kyusyu). Further investigation is needed to explore the moderating effects of areal features when formulating strategies to improve HL in different contexts.

## Supplementary Information

**Additional file 1: Supplementary Table 1.** Health literacy score measured with CCHL in previous studies with Japanese general population.

## Data Availability

The datasets used in this study are not publicly available. However, anonymized datasets may be available after approval by the INFORM study group and the institutional review board. Proposals for use of the data (Research question, Aim, Background, Design, and analytical plan) should be submitted to the corresponding author.

## Abbreviations

CCHL: Communicative and Critical Health Literacy Scale
HL: Health literacy
