# Supplementary material for: Effect modification by geographic area on the association between health literacy and self-rated health: a nationwide cross-sectional study in Japan
Source: BMC Public Health. 2023 May 25;23:952. doi: 10.1186/s12889-023-15897-0 (PMC10209573; doi:10.1186/s12889-023-15897-0)
Supplement: Supplementary file 1 — Additional file 1: Supplementary Table 1. Health literacy score measured with CCHL in previous studies with Japanese general population. [file 12889_2023_15897_MOESM1_ESM.docx]

Supplementary Table 1. Health literacy score measured with CCHL in previous studies with Japanese general population

| Study | Survey year | Target age | N | Mean score | SD | Survey method | Sampling |
| --- | --- | --- | --- | --- | --- | --- | --- |
| This study | 2020 | 20- | 3511 | 3.45 | 0.78 | Mail survey | Two-stage random sampling from all 47 prefectures of Japan |
| Reference [31] | 2017 | 20-79 | 1002 | 3.61 | 0.64 | Mail survey | Randomly selected from a survey research company database |
| Reference [21] | 2014 | 25-74 | 2037 | 3.61 | 0.75 | Mail-placement method | Two-stage random sampling from all 47 prefectures of Japan |
| Reference [32] | 2013 | 20-69 | 713 | 3.59 | 0.62 | Internet survey | Randomly selected from a survey research company database |
| Reference [33] | 2010-2011 | 25-50 | 3663 | 3.63 | 0.64 | Mail-placement method | Randomly selected from the resident registry of four urban and suburban municipalities in the greater Tokyo metropolitan area |
